# Gender Identity and Future Thinking About Parenthood: A Qualitative Analysis of Focus Group Data With Transgender and Non-binary People in the United Kingdom

**DOI:** 10.3389/fpsyg.2020.00865

**Published:** 2020-05-06

**Authors:** Fiona Tasker, Jorge Gato

**Affiliations:** ^1^Department of Psychological Sciences, Birkbeck, University of London, London, United Kingdom; ^2^Faculty of Psychology and Education Sciences, University of Porto, Porto, Portugal

**Keywords:** adoption, assisted reproduction, future parenthood, gender non-conforming, life course theory, thematic analysis, transgender

## Abstract

The idea that people who are transgender or non-binary are not interested in becoming parents has been refuted by several studies. However, both medical unknowns and cisnormativity surround the process of becoming a parent for transgender or non-binary people, with little known about the psychosocial impact on the family formation dilemmas of transgender and non-binary adults. Employing Life Course Theory as our theoretical framework, three focus group interviews were conducted with eleven transgender or non-binary adults. Qualitative data analysis of focus group interview transcripts was conducted through Thematic Analysis. Four overarching interlinked themes were identified concerning the dilemmas perceived by the nine participants who contemplated future parenthood: (i) Balancing a desire for parenthood and desires for other life goals; (ii) Feeling that who I am doesn’t fit into the cisgender system of accessing fostering, adoption or fertility services; (iii) Experiencing the conjoined challenges of gender and fertility embodiment as I see them; (iv) Searching for a non-binary or gender appropriate self and the need for flexible future planning centered on reproductive capacity. Overall, thoughts about gender transition were often interwoven with parenthood plans and in a dialectical fashion the desire and intention to have, or not have, children was implicated in satisfaction with gender transition. The significance of these themes is discussed in relation to how hopes for parenthood could be realized without jeopardizing gender identity and the need for a future focused, flexible, and open-minded approach on the part of fertility and adoption services.

## Introduction

Gender transition has been frequently considered incompatible with parenthood, for example, sterilization is still often considered as a pre-requisite for gender-affirming treatments in many countries (Gunarsson-Payne and Erbenius, 2018). However, empirical studies have consistently revealed that many transgender and gender diverse individuals are already parents (Stotzer et al., 2014). Furthermore, post-gender transition parent−child relationships can be positive and child well-being unaffected, especially in the absence of wider family conflict or stigmatization over parental gender transition (see Freedman et al., 2002; White and Ettner, 2007; Hafford-Letchfield et al., 2019; Zadeh et al., 2019). Notably, studies have shown that a considerable number of those engaged in gender transition desire to have children in the future (De Sutter et al., 2002; Wierckx et al., 2012; von Doussa et al., 2015; Riggs et al., 2016; Cipres et al., 2017; Tornello and Bos, 2017; Marinho et al., 2020).

Transgender and gender diverse individuals are those whose gender is different from that normatively expected from their assigned sex at birth (Riggs et al., 2016; Ellis et al., 2020). While transgender individuals usually have a different gender from the sex they were assigned at birth, those who are gender diverse, non-conforming, genderqueer and/or non-binary take on a questioning or performative stance and hold a fluid conceptualization of gender. Thus, the experience of non-binary or other non-cisgender individuals may be crucially different from that of those who are transgender (Factor and Rothblum, 2008) and this distinction may particularly apply to considerations of parenthood (Stotzer et al., 2014). As the participants in our United Kingdom study primarily identified themselves as either transgender or non-binary we use these terms in the present paper when referring to the participants in our sample and use the terms transgender, non-binary, and gender diverse in reviewing the wider research field.

According to Riggs and Bartholomaeus (2018) previous research on reproduction and parenting has overlooked or subsumed the experiences of non-binary people within a focus predominantly on transgender parenthood. In fact, studies suggest that relative to transgender individuals, non-binary people are both less likely to undertake medical treatments to affirm their gender (Clark et al., 2018) and less likely to receive counseling prior to making decisions regarding fertility preservation (Riggs and Bartholomaeus, 2018).

### Parenting Plans of Transgender and Gender Diverse People

Two pioneering studies concerning the parenting desires of transgender adults found that about half of transgender men (Wierckx et al., 2012) and transgender women (De Sutter et al., 2002) desired a genetically related child. Furthermore, over one third of transgender men said they would have considered cryopreserving gametes had techniques been available previously (Wierckx et al., 2012). Over three quarters of transgender women thought that sperm freezing should be routinely offered before hormonal treatment (De Sutter et al., 2002). However, only half of the participants in De Sutter et al.’s (2002) study indicated that they would have preserved their own gametes had this been possible. More recent studies also have found that while the large majority of transgender individuals agree that fertility preservation should be offered to all transgender and non-binary people, prior to undergoing gender affirming hormonal treatments very few participants actually store gametes (Auer et al., 2018; Riggs and Bartholomaeus, 2018; Marinho et al., 2020). In sum, a low level of fertility preservation among transgender persons is puzzling given the high level of expressed desire for parenthood. However, as most surveys were conducted among attendees of gender clinics (e.g., De Sutter et al., 2002; Wierckx et al., 2012), a further in-depth qualitative investigation with a community sample of those without children may cast light upon the prospective parenthood decision making processes of transgender and non-binary people.

Besides parenthood either through sexual intercourse, via fertility preservation, or via donated gametes to a partner or surrogate, transgender and gender diverse individuals also consider other parenting options, such as adoption or fostering (von Doussa et al., 2015; Nahata et al., 2017; Tornello and Bos, 2017; Marinho et al., 2020). Choices of adoption or fostering appear to be associated with an altruistic desire to help children in need (Tornello and Bos, 2017) and were connected with valuing the formation of socioemotional bonds over and above biological relatedness (Marinho et al., 2020). While a clear picture of preference for genetic parenthood or adoption is yet to emerge, studies to date have indicated that preference rates do differ in different groups. For instance, Chen et al. (2018) reported that 70% of their survey sample of over 150 transgender and non-binary young people considered future parenthood via adoption or foster care. Nevertheless, when genetically related parenthood was considered it was preferred by more non-binary than transgender people. In another United States sample Tornello and Bos (2017) found that transgender women more often expressed a preference for adoption (75%) whereas transgender men were more inclined to seek parenthood through sexual intercourse or pregnancy (58%). Preference rates for future parenthood via fostering or adoption were more evenly split among the Australian transgender and non-binary people in the exploratory survey by Riggs et al. (2016). Over half the sample wanted to pursue biological parenthood (mostly through their partner giving birth) while the remainder planned to explore long-term foster care or adoption.

### Sociodemographic, Psychosocial, and Structural Factors Associated With Transgender People’s Parenthood Decision Making

Prior research has implicated several factors associated with the uptake of fertility preservation and parenthood decision making among transgender and gender diverse individuals including sociodemographic characteristics, psychosocial factors (e.g., personal motivations, family support, narrative resources) and structural barriers (e.g., quality of services and cultural competency of professionals).

#### Sociodemographic Factors

Regarding gender, Auer et al. (2018) investigated the desire for children and the use of fertility preservation options among German transgender women and men in different stages of gender transition. Prior to undergoing gender affirming treatments, transgender men expressed greater desire for parenthood than did transgender women. However, among those who had already initiated treatments, the level of expressed interest in having children in the future was higher among transgender women than transgender men. In Auer et al.’s (2018) most of the transgender men questioned indicated that insemination of a female partner with a sperm from an unrelated donor would be an acceptable route to having children, suggesting that this might be another explanation for transgender men’s relatively low level of interest in oocyte preservation. Consistent with Auer et al.’s findings, other studies have found that transgender women were more likely to undertake fertility preservation than were transgender men (Jones et al., 2016; Chen et al., 2017). The greater complexity of oocyte retrieval and storage for those who were assigned female at birth may account for the fact that transgender men are less prone to preserve their fertility than transgender women. Yet other authors have emphasized the psychologically distressing nature of giving a semen sample, which makes fertility preservation challenging for transgender women (Riggs and Bartholomaeus, 2020, Online First).

Some research teams have found that the level of expressed desire for children and the use of fertility preservation were both particularly low for young transgender young people, even when fertility counseling and fertility preservation options were available (Chen et al., 2017; Nahata et al., 2017; Nahata et al., 2018; Strang et al., 2018). Two other studies have indicated that transgender individuals’ desire to have children may decrease with age (von Doussa et al., 2015; Auer et al., 2018). Reflecting on the difference between the relatively high levels of parenting desire recorded by transgender adults and the low levels of desire (and uptake of fertility preservation) found among transgender youth, Nahata et al. (2017) raised the question as to whether transgender youth might change their perspectives about fertility later in life, particularly after transitioning to their affirmed gender. Strang et al. (2018) also reported that although relatively few transgender youth expressed desire to have their own genetically related child, many speculated or said that they did not know whether their feelings about having a genetically related child could change in the future. Aside from potential discomfort associated with the use of reproductive body parts and gametes that are not embodied in gender identity, Nahata et al. (2017) further speculated that other factors may affect desire for parenthood and contribute to lower rates of fertility preservation utilization among transgender youth, namely, family disruption and rejection and mental health issues (e.g., low self-esteem, depression, self-harm and suicidality).

#### Psychosocial Factors

The psychosocial factors investigated in prior research have involved exploring transgender and gender diverse people’s personal motivations to have children, reporting the extent of social endorsement and support received from within close social networks, and considering how a transgender parent can narratively present themselves to others. Transgender and gender diverse individuals’ motivations for parenthood are quite similar to those of cisgender individuals. These include valuing genetic relatedness and seeking to achieve such relatedness to a child by conceiving of them via intercourse or surrogacy or providing a loving home for a child through adoption (Tornello and Bos, 2017; Marinho et al., 2020). In terms of social support received, support from family of origin has been revealed as an important factor in promoting the well-being of transgender and gender diverse people, including those who are themselves parents (von Doussa et al., 2015; Riggs et al., 2015; Marinho et al., 2020). In fact, in Riggs et al., 2016 study discrimination from family of origin was negatively associated with reports of support for parenting, while support from family of origin was positively associated with the desire of transgender and gender diverse people to have children in the future. Parenting is a highly gender related process within cisheteronormative society and various authors have pointed to the absence of affirmative cultural scripts for transgender parenting (e.g., Haines et al., 2014; von Doussa et al., 2015). Consequently, transgender and gender diverse individuals seeking to become parents have to make sense of and present a coherent psychosocial narrative largely within the mainstream discourses of cisheteronormative societies. In this regard, it was not surprising that participants in von Doussa et al.’s (2015) study tended to shift their narratives between presenting either traditional ideals of heterosexual marriage and parenthood or more radical non-binary approaches to relationships and parenthood.

#### Structural Factors

Aspects that are usually beyond the personal control of transgender and gender diverse individuals when they negotiate parenthood include: (i) obstacles to biological parenting derived from gender affirming treatments and the invasiveness of fertility preservation procedures, (ii) quality of services and cultural competency of professionals, and (iii) the financial costs involved in Assisted Reproduction Techniques (ART).

Transgender and gender diverse individuals who undertake hormonal or surgical gender transition may face specific obstacles that challenge their reproductive capacity and ability to preserve their fertility. Presently, cryopreservation of sperm offers the most viable fertility preservation option for transgender women (De Sutter, 2009; Snyder and Pearse, 2011 in James-Abra et al., 2015). Options available to transgender men who wish to preserve genetic material include cryopreservation of ovarian tissue or more established techniques involving oocyte or embryo storage (James-Abra et al., 2015). However, past research has revealed that transgender individuals perceive these medical procedures as negatively affecting their well-being as these disrupt their gender identity, as participation in them involves sex and gender associated internal or external anatomy (including pregnancy) and interrupts gender affirming treatments (e.g., testosterone usage) that they would rather not delay (Riggs et al., 2015; von Doussa et al., 2015; Armuand et al., 2017; Chen et al., 2017; Nahata et al., 2017; Tornello and Bos, 2017; Petit et al., 2018; Riggs and Bartholomaeus, 2018; Marinho et al., 2020).

Transgender and gender diverse individuals often have to negotiate parenthood options with diverse social institutions such as health and social service providers (Pyne et al., 2015). According to the guidelines published by the Endocrine Society (Hembree et al., 2017), the World Professional Association for Transgender Health (Coleman et al., 2012), and the American Society for Reproductive Medicine (Ethics Committee of the American Society for Reproductive Medicine, 2015) health providers should address potential infertility risk and fertility preservation options with transgender adults and transgender youth and their families before starting gender affirming treatments. While an occasional study of transgender people has revealed both positive and negative experiences within health services (Marinho et al., 2020), most research predominantly reported negative ones (James-Abra et al., 2015; Gunarsson-Payne and Erbenius, 2018; Wingo et al., 2018). These negative encounters in the health care context include having to cope with normative assumptions (e.g., regarding use of gender-related terminology) (James-Abra et al., 2015; Gunarsson-Payne and Erbenius, 2018; Marinho et al., 2020), discriminatory comments (Wingo et al., 2018), and being refused service (James-Abra et al., 2015). Lack of lesbian, gay, bisexual, transgender and queer health competency relevant to reproductive health priorities and treatment also has been reported (Riggs and Bartholomaeus, 2018; Wingo et al., 2018; Marinho et al., 2020). Financial costs are a further factor that might hinder transgender and gender diverse individuals parental projects, especially if public funded fertility preservation procedures are not available (Marinho et al., 2020 in Tornello and Bos, 2017; Riggs and Bartholomaeus, 2018).

### Life Course Theory (LCT)

According to Stotzer et al. (2014) “A more nuanced approach to studying family formation among transgender people will provide better understanding of how transgender people are becoming parents and what their needs may be” (p. 3). Thus, we employed Life Course Theory (LCT) (Elder, 1998; Benson and Elder, 2011) as the guiding theoretical lens for our qualitative research project to consider the subtle and multi-layered contextual influences on personal ideas and decision making regarding gender identity and future family formation with or without children. Life Course Theory has been successfully employed to focus previous qualitative research projects on transgender parenting; for example, Petit et al. (2018) considered both similarities and differences in Canadian pre- and post-gender transition parents.

Five key principles of LCT were considered in forming our research questions (Elder et al., 2003; Allen and Henderson, 2017). First, in LCT all of human development is considered as a life span process (Elder, 1998). Thus, in our study we would expect to see participant’s future thinking about parenthood or remaining childfree reflecting earlier formative or turning point experiences both in childhood and adulthood, notwithstanding that thoughts about parenthood at any one point in time may later change again.

The second LCT concept we considered was cohort: an ever-changing sociohistorical context with regard to both gender transition and decisions about parenthood can be seen to create different social climates for different cohorts of young people making these decisions. The United Kingdom, as elsewhere in the United States, Canada, Australia and Western Europe, has seen a rise in the numbers of young people seeking the help of gender identity services and an increasing differentiation of gender diversity (Twist and de Graff, 2019). Furthermore, emergent adulthood has postponed both partnership and parenthood (Arnett, 2007) which also have been affected by changing socioeconomic circumstances and the increased uptake of college education and training opportunities beyond high school (Côté and Bynner, 2008). Reproductive choice, the need to build up economic resources to provide for children, and later engagement with an increasing variety of fertility services have increasingly characterized entry into parenthood particularly among college educated adults (Umberson et al., 2010; Roberts et al., 2011). These sociohistorical contextual factors can be seen in the decision making of LGB adults too (Goldberg et al., 2012; Bergstrom-Lynch, 2016). Thus, we considered how sociocultural context (cohort and socioeconomic factors) might impact future thinking around parenthood for transgender and gender diverse people.

The third LCT principle we considered was the timing of societal developments in the United Kingdom – specifically regarding biotechnology developments and policy changes with respect to ART and adoption – which may be of greater or lesser significance to any one individual depending upon their chronological age and overall life course agenda. Petit et al. (2018) have highlighted how difficulties in negotiating compatible services have differentially affected distinct cohorts of pre- and post- transition parents depending upon their individual biographies.

In the United Kingdom, as elsewhere in the United States, Canada, Australia and Western Europe, the landscape of parenthood possibilities for transgender and gender diverse people has been changed by developments in medical knowledge and practice around gender transition and in assisted reproduction (Golombok, 2015; Wylie et al., 2016; Condat et al., 2018; Baram et al., 2019). Condat et al. (2018) draw attention to the ethical aspects involved in biotechnologies that facilitate transgender individuals access to parenthood, both at individual (e.g., effects of hormone suppression) and social (e.g., challenging conservative norms) levels. This way, taking into account ethical principles of beneficence and non-maleficence, autonomy, and justice (Beauchamp and Childress, 2013), these authors consider that while technical advances allow transgender persons to self-actualize as individuals, partners, spouses and parents, research on these issues should nevertheless continue. With regard to accessing ART, research studies with Australian transgender and non-binary people (Bartholomaeus and Riggs, 2020) and healthcare professionals (Riggs and Bartholomaeus, 2020, Online First) have pointed to the role of healthcare professionals, not only in providing information, but also in gatekeeping access to fertility preservation either by pushing a pronatalist fertility preservation agenda or by implicitly or explicitly placing obstacles. In the United Kingdom funding decisions concerning publically funded National Health Service (NHS) provision for fertility preservation procedures are made regionally and at present there are no national guidelines on providing fertility treatments and storage for transgender or non-binary people (Human Fertilisation and Embryology Authority, 2020).

Parenthood possibilities also have expanded through the opening up of both fostering and adoption to same-gender couples (Mallon, 2011; Brown et al., 2015). However, foster care and adoption agencies generally have been slow to recognize the rights of transgender and gender diverse people to be assessed as potential parents, such that transgender people who wish to adopt may experience discrimination in these services (Stotzer et al., 2014; Riggs et al., 2016; Tornello and Bos, 2017). In the United Kingdom legislative change has opened up adoption to lesbian and gay couples but placement rates have remained low (Tasker and Bellamy, 2019) and services have been slow to consider transgender people as potential adoptive parents or foster care providers (Brown et al., 2018; Brown and Rogers, 2020).

The fourth LCT concept that we have considered is the perception of human agency. Life course theory considers that agency or “free will” can operate within the limits of the social and cultural world as this is interpreted and re-interpreted by the individual over time: “within the constraints of their world, people are planful and make choices among options that construct their life course” (Elder, 1994 p. 6). Unlike most cisgender people, transgender and gender diverse people may well become gradually aware of various potential obstacles to gender identity fulfilment and future parenthood early on in life and over their life course actively make plans to navigate around these. We expected that participants in our focus group study would be keen to tap into other transgender and non-binary people’s knowledge about the implications of a childfree lifestyle or about future parenthood options via fertility preservation or adoption.

The fifth LCT concept we considered in relation to future thinking about parenthood concerned the importance of social connections established and maintained with others (i.e., the role of linked lives in experiences) particularly with respect to family of origin and partnership (Wong, 2018). Thus, for our participants we anticipated that both gender transition and parenthood plans may also be impeded or assisted by significant other people in their lives, such as, considerations with respect to family of origin. For example, Riggs and Bartholomaeus (2020, Online First) found that while some parents seemed to support the decisions the young transgender or non-binary person themselves made concerning accessing hormonal and surgical procedures and their choices about fertility preservation, other parents acknowledged either insisting or encouraging their child to do so. Further, perceived obstacles to having or not having children (and the personal choices made regarding these obstacles) are also likely to be influenced by partnership choices and a partner’s potential reproductive capacity (Petit et al., 2018).

### Research Aims

In our exploratory study we aimed to sample a range of views and rationales within the transgender and non-binary community as to whether parenthood was desired or whether participants would prefer to remain childfree. If parenthood was considered, we also wanted to examine the routes to parenthood (via ART or via adoption and fostering) that participants desired and thought to be possible. Previously, the careful and sensitive juxtaposition of different views about parenthood and routes to parenthood within a single study have highlighted common or distinct positions within a sexual or gender minority sample, as seen for example in Bergstrom-Lynch’s wide ranging study of childfree and LGB parents (Bergstrom-Lynch, 2016; Tasker, 2020). Therefore, we judged data collection via focus group interviews within the context of a community group setting to be a useful method for gathering a range of viewpoints from transgender and gender diverse groups. Focus group methodology also had the added benefit of providing direct opportunities for community empowerment via the interchange of knowledge and experience at the point of data collection (Krueger and Casey, 2015; Wilkinson, 1999).

## Materials and Methods

### Participants

A total of 11 participants contributed to the discussion in one or two of the three focus groups organized: seven participants contributed to one focus group and four participants attended two focus groups. None of the participants were living in the gender they had been assigned at birth. Participants described themselves in the following ways on a brief demographic questionnaire: four participants identified as men or as transgender men; one participant identified sometimes as a man and sometimes as non-binary; two participants identified as women or as transgender women; four participants identified as non-binary. Eight participants had undergone hormone therapy at the time of the study and seven participants had received upper (chest) surgery but only two participants had undergone both upper and lower (genital) surgery.

Participants ages ranged between 20 to 45 years old: seven were aged between 20 and 29 years while two were aged between 30 and 39 and two were 40 years plus. All participants were living in the United Kingdom, residing in and around the London area at the time of data collection. All participants except for one identified as white English or Irish. Eight participants reported no disabilities. Three participants reported having a mild level of disability with an effectively managed impact on daily life (these included dyslexia, mild ADHD, and issues related to anxiety). Regarding professional occupations, five were undergraduate students and the remaining worked in the following areas: teaching or academic, care or customer related, external relations or information technology. One participant did not report an occupation. As for annual income level, two participants declined to disclose information, two reported no income, two participants reported incomes of £10,400 up to £15,999, one participant reported £26,000 up to £31,199, three participants reported £31,200 to 36,399 and one participant reported £46,000 up to £51,999.

### Procedure

Participants were recruited through Gendered Intelligence a charitable organization based in London in the United Kingdom, which was established as a Community Interest Company in 2008. Gendered Intelligence’s mission aims to increase awareness and understanding of gender diversity and works with the transgender community with a particular focus on young people’s needs. Through the authors’ prior discussions with Gendered Intelligence a common interest had been established in the need for more research into the views and experiences of people on the transgender spectrum in relation to future parenthood. Thus, the focus of data collection was on hearing the viewpoints of transgender and non-binary people concerning fertility and parenthood, whether or not parenthood was desired.

Recruitment to the focus groups was mostly done through Gendered Intelligence. Staff at Gendered Intelligence electronically mailed out an advert to their online mailing list inviting transgender and non-binary people to contribute to research-based focus group discussions run by the authors, who were identified by their university affiliations. Initial details mailed out included the dates for the first two groups and the venue. Additional publicity was distributed through the authors’ networks and those who received the initial information were asked to distribute publicity materials within their own networks. The number of people who received the introductory distribution was unknown, thus in common with other studies employing convenience sampling techniques we have no method of calculating a response rate or the reasons for non-response (Jager et al., 2017). Recipients could then request further details about the research from either Gendered Intelligence, or by contacting the authors, and were sent an information sheet about the project, the main questions to be addressed in the focus group (as specified below), research consent forms, and brief academic biographies of the authors.

Three inclusion criteria were employed in establishing eligibility for focus group participation: participants had to be 18 years old or more, be transgender or non-binary, and not already have genetically related children or children who lived with them. The main questions tabled for group discussion were as follows and interviewers encouraged participants to expand upon their answers: Have you thought about becoming a parent? Have you thought about different ways of becoming a parent? Have you decided not to become a parent? What are the most important aspects involved in bringing up a child? Is partnership important for parenting? What are society’s views on queer or transgender parenting? What would be your family’s views on queer or transgender parenting?

For the convenience of participants focus group discussions were held at the central London premises of Gendered Intelligence. The first two focus groups were held in February 2016 (one in the afternoon at the weekend and the other on a week-day evening). Four participants attended the first focus group and seven people participated in the second focus group discussion (including one person who had previously attended the first group and additionally wanted to attend the second). Each of these focus groups lasted approximately 2 h.

At the start of the focus group participants were handed the information sheets about the research, consent forms, and the brief demographic questionnaires that yielded the sample details given above. The authors also verbally briefed those attending the focus group on the information sheets and consent forms at the start of each focus group. The briefing included a discussion of the ground-rules for the focus group discussion to ensure that participants were respectful and supportive regarding different views or gender positions and that any identifying information shared during the discussion was kept confidential within the focus group (Breen, 2006). Participants also were invited to say as much or as little as they felt comfortable with and reminded that they were able to leave the discussion at any point if they wanted to do so (one of the focus group interviewers was ready to individually debrief a participant if this had occurred). As interviewers we were mindful of the balance between the risks of over-disclosure in a group setting versus facilitating supportive discussion (see Sim and Waterfield, 2019). Participants were told that they should choose a pseudonym with which they should identify themselves at the start of the discussion and give their preferred pronouns. Pseudonyms have been re-assigned in the transcript extracts presented below. For the ease of assigning speakers during the transcription process, we also requested that each participant add in a neutral piece of information, such as a favorite food, color, plant or animal together with a reason why they liked it. Prior to the start of the audio recorded discussion, participants were asked to sign their consent form and to complete a quick questionnaire to give demographic details. Participants also were told that they had the opportunity to review their consent to their data being included in the research at the end of the discussion and were informed that they had a further 2-week period during which they could withhold their individual data from the focus group transcript by contacting the authors. Thus, all participants gave their informed consent and none withdrew from the study either during an interview session or subsequently.

A third focus group was conducted in September 2017, using the same procedure as the initial two focus groups. The purpose of the third focus group was largely to facilitate thematic verification and then further refine the themes with additional information or comments. Participants were shown the researcher derived subthemes and themes from the first two focus groups and invited to discuss them. For the third focus group we specifically invited those who attended the first two focus groups and also welcomed comments from any new participants, who had inquired about the study, met criteria for participation, and wanted to attend. The third focus group was approximately 1.5 h in length. The third focus group began with participants reviewing the list of twelve preliminary themes and subthemes generated from the thematic analysis of the data from the two initial focus groups.

The entire procedure for the study was approved by an Institutional Review Board. All three focus groups were transcribed by a professional transcriber and the transcripts then checked by the first author. In the verbatim transcript extracts that appear below minor edits have been made to preserve confidentiality, condense length, and improve readability.

### Thematic Analysis

Qualitative analyses of interview data were conducted using the Thematic Analysis (TA) approach delineated by Braun and Clarke (2006, 2013). The main focus of TA was to identify meaningful patterns within the data not only to summarize content but also to elucidate the overall meaning that participants sought to convey.

In the first phase of the analysis the first author open-coded data from the initial two focus groups. Specifically, each focus group transcript was read several times by the first author, who then began the process of open-coding the data. Boyatzis explained the qualitative process of open-coding as noting: “the most basic segment, or element, of the raw data or information that can be assessed in a meaningful way regarding the phenomenon” (Boyatzis, 1998, p. 63). These open-codes were then reviewed *in situ* on the transcripts of the initial focus groups by the second author who made modifications and additions in discussion with the first author. The first author then grouped the agreed upon open-codes in terms of their perceived similarity and difference and labeled each grouping, either nominating an existing open code as a sub-theme exemplifying the grouping or by writing a new sub-theme label. The first author then grouped and re-grouped sub-themes together in terms of how these cohered into themes (which conveyed a meaningful interpretation of different facets or aspects of the focus group data). After undertaking a review process involving further iterations of the subthemes, the list of themes and subthemes together with their contributing open codes were shared with the second author and thus reviewed again to derive twelve preliminary themes.

In the second phase of analysis we employed two different approaches and techniques to audit and then further refine the initial set of twelve preliminary themes (Burnard et al., 2008). One technique involved participant or member checking (Morse et al., 2002; Birt et al., 2016). Here, we recognized that the original focus groups could not be re-created at a later point, or possibly even in practical terms reconvened, to establish the veracity of themes. Furthermore, we acknowledged that member-checking has been critically evaluated by some qualitative researchers on epistemological grounds (e.g., Ashworth, 1993). Therefore, we sought to establish the credibility of our qualitative findings in different ways (Yilmaz, 2013). We adapted Birt et al’s synthesized member checking procedure to our focus group setting keeping in mind the particular ethical constraints of confidentiality in relation to the original focus group generated data (transcript). Thus, participants in the third focus group reviewed and commented upon the twelve themes and associated subthemes generated from the analysis of data from the first and second focus groups. After giving their initial endorsement of the twelve themes, participants in the third focus group then further discussed these themes in relation to their own experiences. After reviewing the transcript from focus group three the authors retained the twelve themes with only minor modifications.

The second technique was deployed to establish the credibility of our qualitative findings via independent audit. In this audit the twelve preliminary themes were used as a focused coding framework (Charmaz, 2006) for a fresh analysis of the original transcript data from the first and second focus groups in a secondary analysis by an undergraduate student research assistant, who had not been involved in research design or data collection. When the same transcript extract was coded under the same theme in both the initial and secondary data analyses the theme was seen to be independently endorsed. Then the nine themes that had received independent endorsement were used in the focused coding of data generated by the third focus group (again completed by the undergraduate student research assistant). After peer review and discussion between the authors, one theme was split into two and these ten themes were re-grouped together under the four overarching themes detailed below.

## Results

Thematic Analysis of interview data generated four overarching themes across all three focus group discussions: Balancing a desire for parenthood and desire for other life goals; feeling that who I am doesn’t fit into the cisgender system of accessing fostering, adoption, or fertility services; experiencing the conjoined challenges of gender and fertility embodiment as I see them; searching for a non-binary or gender appropriate self and the need for flexible future planning centered on reproductive capacity (see Table 1).

**TABLE 1:** Overarching themes and contributing themes from thematic analysis of focus group data.

Overarching theme	Contributing themes
Balancing a desire for parenthood and desires for other life goals	(a) Is having children a priority worth sacrificing other life goals for?
	(b) Desire to have children but need to get ready to have children
	(c) Diverse family forms can support parenthood, but which suits me?
	(d) Having support from extended family is important for deciding to have children, especially if no partner, but if you do not have it, you just plan and get on with it
Feeling that who I am doesn’t fit into the cisgender system of accessing fostering, adoption or fertility services	(a) If you don’t conform to the gender binary then parenting is a social challenge but not necessarily a medical one
	(b) But if you’re *trans* it’s relatively straightforward socially but complicated medically and often blocked by ignorance and/or prejudice
Experiencing the conjoined challenges of gender and fertility embodiment as I see them	(a) Problem with lack of biological fertility for appropriate parenthood is that this challenges to your non-cisgender sense of self
	(b) The opportunity to preserve own fertility is worth having, aside from whether or not you ultimately have a baby
Searching for a non-binary or gender appropriate self and the need for flexible future planning centered on reproductive capacity	(a) Worth keeping fertility under review, because feeling happier with your gender makes you feel more like pursuing life goals like parenthood
	(b) Taking a pragmatic approach: avoid reading reproductive parts as gender parts, but that’s really difficult to do when others misread them

### Balancing a Desire for Parenthood and Desire for Other Life Goals

Of the 11 people attending the focus groups two participants stated that they were committed to remaining childfree, although one of these participants identified as a stepparent to their partner’s adolescent and young adult offspring who did not live in their home. For example, Stephen said that becoming a parent had never really appealed as he humorously commented: “I somehow feel like I kind of missed the class at school, you know, where they go into [it] and they say okay, you know, human beings are attracted to each other and then some of them reproduce!” (FG2 line 419). Stephen also elaborated upon the practical downsides of having young children who would not be able to fit in with his lifestyle. Stephen explained that his decision to remain childfree was not to do with being transgender, but something he probably would have done anyway:

“and to me the practical thought of, you know, when I go home at the end of the evening I’m knackered … and the thought of having little people, you know, tugging at my legs, or whatever, and me having to, you know, get up early in the morning. It just is really not attractive. And I was beginning to think is it due to [me] being *trans* or not? And I’ve come to the conclusion it’s probably not, um, that, you know, if I wasn’t I’d probably still. you know, feel the same way.” (Stephen FG2 lines 430−443).

The remaining nine participants were to varying degrees potentially interested in becoming a parent in the near to distant future, but some also seemed somewhat cautious and concerned: “I’ve always kind of wanted to be a parent, er wanted to be to a scary extent. Um, yes, it’s always been kind of you know always been my motivation to do anything” (Mars, FG2, line 485). One aspect of being cautious was seen in concerns participants divulged about having certain prerequisites in place for parenthood, such as being financially prepared, having suitable living accommodation for a child, or having a career path ahead: “I was like ‘Oh my God, I really want babies! (laughs) So maybe I should do it now?’ But then I realized that, actually I started to think about more important things, like then I hadn’t started my career and stuff, so the emotions died down” (Seth FG2, line 675). Other participants wondered as Ethan did about the enormity of taking on responsibility for a child’s life and whether personally were up to the challenge: “it’s just such a massive decision, and such a life changing decision, and um have I really got it in me to become a parent and become a dad?” (Ethan FG2, line 114).

Participants did not necessarily see partnership as a prerequisite to parenthood, although views on this varied. The participants in Focus Group 1 seemed to agree tacitly with Rain who said: “I think the probably the best way for children to grow up is to have a whole range of people that they are close to […] You have that whole range of experience and background um and positive influence from people.” (Rain, FG1, line 684). In Focus Group 2 Seth also said: “The idea of having a nuclear family with two parents is no longer – it doesn’t really feel like that’s actually important. We’re just told it is.” (Seth, FG2 line 800). In contrast, Ethan stated that in terms of his own situation: “I’ve always been clear that I don’t want to be a single parent, neither biologically, or through other means” (Ethan FG2, line 1490). For Ethan parenthood was connected to realizing his gender identity (thinking of himself as a dad) and then made possible through partnership and his partner’s desire to become a mum:

“In my mind’s eye I think I’ve always had a fantasy of having a family and seeing myself as a parent, and specifically of being a dad, but it’s been kind of wishful thinking and I never thought I would come close to it becoming a real potential. But since falling in love with a woman, who is very keen to become a mum, [then] I think one reason why I’ve decided to come to this focus group is to try and articulate these thoughts in my mind.” (Ethan, FG2, line 0089).

Having at least some extended family members who endorsed participants’ plans for parenthood was perceived as helpful and supportive, but not necessarily a decisive factor. Seth said:

“[My mum’s] just that kind of person that thinks having babies is so cool! [laughs of appreciation from other FG members]. That was a really positive influence and I kind of always knew that she’d be okay with it. And when I had that little freak out before going on T [testosterone], whether I should do this now or -, she was right there saying if you do want to then that’s fine I will help you, you know, whatever that entails. If I didn’t have a partner she would help with care all that sort of thing, pretty much anything. So yeah kind of practical helping, but also just never questioning, never sort of saying to me well if you feel this way [i.e., wanting to have a baby], you know, does that mean that you’re not really-? You know, those kind of questions that I think a lot of people would think if they didn’t know what it’s like to be trans, I guess. But the rest of my family aren’t necessarily supportive. I haven’t actually told them, or spoken to them, about it.” (Seth, FG2, line 0749).

### Feeling That Who I Am Doesn’t Fit Into the Cisgender System of Accessing Fostering, Adoption, or Fertility Services

For focus group participants who identified as non-binary future parenting presented a psychosocial challenge in terms of the social and cultural issues they faced in reaching future parenthood, yet often achieving parenthood was less complicated medically. As Rain explained in the interview quotes below: how would they identify themselves as a parent? If they applied to foster or adopt were there additional legal obstacles that they would face? However, for Rain and their partner, becoming a co-parent could be relatively easily accomplished by following the well-trodden pathway with their female partner’s donor insemination.

“My partner’s biologically female and I’m biologically female, so well (…) I assumed because I identified as gender queer and wasn’t planning on like physically transitioning and undergoing hormone therapy, at least not at present, that it would just be seen more like well essentially a lesbian relationship and go through that process of like having children [i.e., with donor sperm insemination], which would potentially be easier. But obviously I’m not entirely happy with that because I don’t identify as female I identify as gender queer and gender fluid, so I guess for me it’s well less the physical situation and more the social situation.” (Rain FG1, line 0169).

As Rain explained further later in the focus group discussion:

“Because I don’t identify as um female, my [biologically female] partner (…) kind of just assumed that she would be the one who would be the biological mother if we went down that route [to parenthood]. So, um, yeah, but there’s still the social issue of well, what would I call myself as a parent? I’d be happy with just being a parent, or just come up with a new word for parent, um, so not mom or dad, but something else entirely. Um and then in terms of [adoption] and fostering, I don’t know if there’s additional legal stuff to work around, yeah, if you’re *trans* and wanting to adopt but non-binary.” (Rain, FG1 line 0410).

Other participants pointed to societal barriers to parenthood highlighting the likelihood of encountering prejudice or ignorance when applying to foster or adopt children. For example, Pete drew contrasts between his perception that United Kingdom adoption services had become accepting of cisgender same-gender couples, while a transgender same-gender couple or a queer family would press at and likely exceed these boundaries.

“There’s less of a sort of stigma about kids being adopted by gay couples, but I think with *trans* people there’s still that sort of suspicion. I mean it’s further complicated sort of by, you know, if you’ve got a nice you know male/female couple and like one of them happens to be *trans* then that’s more or less okay. But when you get into sort of queerer families or one of them being non-binary then it’s just -, you know, the people who set kids up with foster parents don’t want anything to be controversial” (Pete, FG1 line 432).

Nevertheless, concerns about the likelihood of an application to foster or adopt being accepted were not the only reasons given for not pursuing adoption or foster care. This route to parenthood raised further doubts for some participants who were concerned that any future child would be affected negatively by the legacy of foster care or adoption. Toyah said: “I always assumed that fostering was almost identical to adoption, except that maybe the child was in a rough situation. […] But that’s really why I would avoid fostering personally because I see it as something where that’s more of a challenge to do it.” (Toyah, FG1 line 403). While Pete pointed out the potential for extended family members to be less accepting of an adopted child than one who had a genetic connection: “My mum’s quite anti-adoption, she just thinks that every child that’s up for adoption is just going to end up some crazy mess!” (Pete, FG1 line 1372).

Much of the focus group discussion and interchange of information focused upon accessing appropriate fertility services. While none of the transgender or non-binary people who had been assigned female at birth had undertaken oocyte storage, sperm storage had been successfully carried out by Kim one of the transgender women. The dilemmas for transgender men and transgender women were different because of the distinctly different roles played by female and male reproductive organs. Transgender men could only preserve the capacity for genetic and gestational parenthood by retaining their uterus, the return of menstruation and/or oocyte collection for cyropreservation (involving coming off testosterone supplements and artificially boosting undesired estrogen levels). Thus, both of these factors presented transgender men with surgical intervention and the expense of this. For transgender women, sperm retrieval was often expected to be through the ejaculation of a semen sample, which presented transgender women with considerable psychological challenges around embodiment. No one in the focus groups mentioned the possibility of the surgically aspirating sperm directly from the body. Both transgender men and transgender women faced potential hormonal treatment disruption and financial expense for gamete storage.

Several of the conflicting issues involved in the challenge of accessing fertility preservation services were voiced by Pete. Pete’s preferred route to parenthood would be first to cryopreserve his eggs, subsequently to use *in vitro* fertilization with his male partner’s sperm, and then to have a surrogate carry the pregnancy. Pete found contemplating all this quite stressful: “I have to think of it now because in terms of surgery and hormones and stuff, it’s forcing me to make [fertility] decisions now [about egg storage] that most people don’t have to make until they’re much older.” (Pete FG1, line 0066). Pete also thought that his choices had been severely limited by a combination of ignorance and prejudice on the part of health professionals.

I’ve actually been to fertility clinics and done all the testing and stuff and I didn’t actually get the funding for it, just because [their guidelines] on transgender patients and egg freezing and that sort of stuff are a bit blurred. They said because, erm, currently [commercial] surrogacy isn’t legal in the United Kingdom and I don’t have a willing surrogate right here now, they weren’t going to do it. Erm but I was kind of with the view that you’re [planning on] taking them out, so, like why? Like I didn’t quite understand it, because if I was a cancer patient you would store them. Because it’s not really that different because a cancer patient is not going to be able to carry her own eggs. So, yeah, I was a little bit-, I thought well that was […] well transphobic really. (Pete, FG1 line 0095).

Transgender participants also were faced with health care professionals’ assumptions that being transgender meant either not wanting to have children or forgoing parenthood for gender enhancement. One consequence of this was that not only was hormonal support for gender identity withdrawn while conception and pregnancy were pursued, but also simply initiating a discussion about pursuing fertility treatment could mean risking the loss of psychological support too. Phil told Focus Group 3:

More recently when I told the consultant at the gender clinic that I was planning on coming off T [testosterone] to try and conceive, he was really shocked because I think he thought that I was this kind of classic *trans* man and I was like professionally successful and ticked all these boxes and well I just shattered all his illusions [Group laughs] like he’s got really weird ideas about stuff! And then he said [that] because I wasn’t pursuing surgery at that point he was going to discharge me from the gender clinic. I felt like I don’t know if that’s the right thing right now. And like a year later I can definitely say it wasn’t the right thing, because I could have done with some counseling, some support from the gender clinic (Phil, FG3 line 0617).

In summary, focus group discussions often indicated the lack of fit between the varied needs of transgender and non-binary people as they sought parenthood and the systems set up to assist cisgender people to achieve parenthood. Nonetheless, some participants were hopeful that at least discussions about fertility options were starting to happen. Stephen, who was happy living a childfree life, said: “When I started my transition it was never even put to me. You know I was just told: if you want to transition then start testosterone. But well it sounds like people who have transitioned a bit more recently are beginning to have these conversations with medical professionals, you know, hopefully” (Stephen, FG2 line 1875). And as Kim said: “Nothing was explained. I had to do my own research, yeah, I had to fight hard, but I finally got there [sperm storage] and it was worth it!” (FG2, line 0280).

### Experiencing the Conjoined Challenges of Gender and Fertility Embodiment as I See Them

For transgender participants in particular attaining biological parenthood was complicated because reproduction emphasized the presence of body-parts that contradicted gender identity. The challenging clash between fertility embodiment and gender were movingly voiced by Pete. Pete identified as a gay man with a cisgender gay partner and explained his discomfort in the following dialog:

Pete: I identify as a gay man, so technically we could have like a child in the normal way, but I would never carry a child, because that would -, that’s weird to me because I’m not female. So yeah that’s something I would never do.

Interviewer: So carrying a child would feel like –

Pete: That would be weird to me

Interviewer: A woman’s bit?

Pete: Yeah it’s just something that I don’t feel comfortable with

(FG1 line 0214)

In a parallel fashion, making difficult decisions affecting future fertility opportunities was further complicated by the urgency of making progress with gender-appropriate hormone supplements to assist gender embodiment. Ocean’s conversation with Pete illustrated the psychological experience of pressure to postpone egg storage in favor of going on testosterone. The lack of medical clarity about the effects of taking testosterone supplements on the viability of oocytes further added to the complexity of how to manage what appeared to be competing priorities.

Ocean: When I went on hormones when I think I was like 19 or 20 um they [medical professionals] said “Do you want to stash your eggs?” and explained that you have to go on estrogen hormones for a while and then it would take ages. I said no I need to get the testosterone in me, so it was a snap decision, but one that yeah that’s going to have like quite a lot of consequences though.

Pete: I mean you can go back from that. I mean just because you’re taking testosterone doesn’t mean that you can’t …

Ocean: Yeah, but I think it makes it sort of riskier with the eggs and so on

Pete: They’ve said to me that it’s fine (FG1, line 0076).

### Searching for a Non-binary or Gender Appropriate Self and the Need for Flexible Future Planning Centered on Reproductive Capacity

The nine participants who were keen to explore the idea of becoming a parent in the future had previously had at least some earlier thoughts about becoming a parent when they themselves were still at school. Nevertheless, during the focus groups participants described how their thoughts about becoming a parent in the future came and went over time. As seen in Ocean’s quotes above, and in Ethan’s extract below, participants put thoughts of parenthood aside in favor of accessing hormonal supplements when discomfort with assigned gender peaked. However, actively wanting to pursue parenthood seemed to be prompted by feeling happier about achieving an appropriate gender or non-binary sense of self. Thus, some participants felt caught in a paradox of feeling psychologically ready for parenthood yet further away from attaining biological parenthood. Ethan explained the dilemma:

So now that I’m much more, erm, at ease in my body and can barely remember the anguish of pre-T, erm, I have regret: Why didn’t I do it? [egg storage]. But I just need to try and remember how awful that felt. I immediately know that I just couldn’t have done it. But it would be nice now to have. But it was just mentally -, it was never a possibility. I just could not have entertained that. (Ethan, FG2 line 976).

One solution put forward in Focus Group 2 was to take a pragmatic approach to having a baby: use available reproductive body parts without thinking of these as embodying gender. Nevertheless, as Seth expanded upon the idea of taking a pragmatic approach it became clear that difficulties could be potentially posed when pregnancy became visible since others could start to misinterpret gender causing personal anguish.

“Pregnancy itself doesn’t feel inherently female anymore. I’m at a point now I’ve thought about it so long and so hard. I’ve always wanted kids and I’ve never identified as female, so for me having kids isn’t a female thing it’s a mechanical thing – they just aren’t tied to each other. But if I start to be read as female then it’s going to mess with my head […] you know I’ll just go and hide or something for 9 months!” [focus group laughs] (Seth, FG2 line 0712).

## Discussion

To a greater or lesser extent parenthood was clearly part of a future life plan for most of transgender and non-binary people who participated in our focus group interviews (De Sutter et al., 2002; Wierckx et al., 2012; von Doussa et al., 2015; Riggs et al., 2016; Cipres et al., 2017; Tornello and Bos, 2017; Marinho et al., 2020). From accessing various online resources our participants were knowledgeable about fertility possibilities after beginning hormonal or surgical gender transition and in some cases participants said that they were informing the health care professionals with whom they came into contact (Twist and de Graff, 2019). In our study gender identity fulfilment and parenthood aspirations often appeared to be complexly interwoven: childhood fantasies about future parenting may have alerted a young person to their gender identity; the need to make progress with gender transition may have put thoughts of parenthood on hold; attaining comfort with gender identity could promote the desire to become a parent.

Other authors employing an LCT framework have noted the reciprocity of gender transition appreciation and parenthood decisions when interviewing transgender pre- and post-transition parents. For instance, Petit et al. (2018) noted that gender transition appreciation was an integral part of a life course agenda concerning decisions to have or not to have children and likewise thoughts about future parenthood in turn informed the process of achieving comfort in a transgender or non-binary identity. In a similar fashion in our study, the first theme – the balancing of a desire for parenthood or not having children and the desires for gender identity fulfilment and other life goals – was derived from qualitative data on the perspective of transgender and non-binary adults who do not have children but who were making decisions about future parenthood or remaining childfree and who also were sometimes simultaneously deciding upon hormonal and surgical interventions to assist gender presentation. Previous prospective parenthood studies of transgender people’s views have not been framed explicitly within a developmentally focused LCT framework and generally have not considered the particular perspective of non-binary people. While our investigation of development has been hampered by a cross-sectional approach and also by our small sample size, like Petit et al. (2018) we also found evidence of changing views on having children and on gender related processes over time.

As Petit et al. (2018) found the LCT concept of human agency with respect to decision making and future goals played a crucial role. In practice, for the participants in our study this meant that if parenthood was desired then a key aspect was also developing a flexible future plan to run alongside a quest for a non-binary or gender appropriate self (theme four).

Nonetheless, parenthood was seen as a daunting project. While adoptive parenting was rarely ruled out completely (von Doussa et al., 2015; Nahata et al., 2017; Tornello and Bos, 2017; Marinho et al., 2020), participants judged that applications to adopt made by transgender or non-binary people would be very unlikely to succeed. Although the United Kingdom has been at the forefront of legislative change to allow same-gender couples to adopt, adoption is still a contended topic (Tasker and Bellamy, 2019) and opening up foster care and adoption to transgender and non-binary applicants is only just beginning (Brown and Rogers, 2020). In relation to LCT, the current sociohistorical context in the United Kingdom thus favored consideration of fertility preservation upon which focus group participants had already garnered knowledge. Furthermore, older participants in our focus groups noted that younger participants were having conversations with health care professionals that they themselves had not had, thus highlighting the importance of the LCT concept of timing (age and life course agenda) in relation to contextual changes. Discussions in all three focus groups concentrated on biological parenthood via ART, but as other authors have noted professional “gatekeeping” pertained here too (Riggs and Bartholomaeus, 2020 Online First). Our participants were faced with a contradictory series of service gateways: some gateways were beginning to open up to fertility preservation (gender clinic services). But participants might then find further gateways closed, perhaps through lack of personal finance to circumvent the absence of designated transgender and non-binary appropriate state funding at ART clinics. Thus, the underlying theme that echoed as a refrain through the conversations was one of our participants not feeling able to present a good enough fit to unlock the cisgender or binary social systems that governed services (theme two).

In contemplating genetically related parenthood, transgender and non-binary people were faced with uncomfortable reminders of the reproductive organs and gametes associated with their birth-assigned sex. In turn these reminders raised concerns about being able to realize biological parenthood without jeopardizing the security of the gender identity position that participants had worked so extremely hard to attain (Riggs et al., 2015; von Doussa et al., 2015; Armuand et al., 2017; Chen et al., 2017; Nahata et al., 2017; Tornello and Bos, 2017; Petit et al., 2018; Riggs and Bartholomaeus, 2018; Marinho et al., 2020). Participants particularly anticipated the reactions of other people to their fertility: would others read them as a father-to-be if they were carrying a child? Would others read them as a mother-to-be if they provided the sperm and were not pregnant? Hence our underlying theme of gender and fertility embodiment challenges (theme three). These thoughts that interlinked twin concerns of gender identity and fertility substantially added to the usual concerns also experienced by cisgender people undergoing ART, namely, anxious uncertainty about the chances of successfully having a baby (Purewal et al., 2018), the physical and psychological challenges of the procedures (Moura-Ramos et al., 2012; Dornelles et al., 2016) and the financial costs of ART in the United Kingdom as in many countries (Culley et al., 2011). Nevertheless, despite these multiple challenges to achieving parenthood, our transgender and non-binary participants spoke of the psychological value of preserving fertility possibilities even if these were not activated in the future.

Participants framed their decision making around having children or remaining childfree within the personal context of their own life story: Was parenthood desired? And if parenthood was sought after, could parenthood be accommodated sooner or later within their life course? The two participants who had decided not to have children thus framed their decision in terms of never seriously wanting to have their *own* children and being satisfied with their existing relationships with children in their networks. For example, participants thought of themselves as stepparents to their partner’s children, described avuncular but gender−neutral relationships with their siblings’ children (niblings), and/or had worked in a paid or voluntary capacity with children. None of our sample wanted a completely childfree life.

The nine participants who to a greater or lesser extent placed a priority upon parenthood for themselves saw the desire for parenthood as an evolving part of their overall life course story that was intimately connected with their gender journey (Petit et al., 2018). For some an important aspect of recognizing their gender identity during childhood or adolescence had been the reflection that they wanted to be a mother or a father or simply a parent and specifically not a parent of the gender they had been assigned at birth. Nevertheless, other participants recounted that the desire to parent preceded gender questioning and was independent from it, except that hormonal or surgical plans to assist gender transition might impinge upon fertility.

Participants foregrounded concerns regarding their own fertility over other routes to parenthood within the focus group discussions. Both transgender and non-binary participants varied in their commitment to having children who were genetically related to them, not only because of their own desire for progeny, but also because of the perceived societal and social obstacles they anticipated encountering on other routes to parenthood. When interviewers specifically asked about adoption, focus group participants indicated that they thought it unlikely that adoption services would support an application to adopt made by a transgender or non-binary person. Previously authors such as Bergstrom-Lynch have pointed to the more affirmative assisted reproduction service based approach conducive to the LGB couples (comprising mostly of cisgender individuals) that Bergstrom-Lynch interviewed and contrasted this with the (hetero)normative lens of providing a family life for children in need that has characterized adoption agencies in the United States (Bergstrom-Lynch, 2016). Thus, our participants also perhaps judged that commercially driven fertility services would be more open to their inquiries than would statutory adoption services in the United Kingdom. Some of our participants expressed additional concerns that an adopted child would potentially have to deal with the double challenge of societal prejudices against both adoption and having a transgender or non-binary parent, potentially on top of placement in a same-gender couple headed household when a participant did not identify as heterosexual.

Congruent with findings from studies that have focused on sexual identity (Stacey, 2006; Roberts et al., 2011; Goldberg et al., 2012; Bergstrom-Lynch, 2016) partnership sometimes contextualized parenthood plans for our transgender and non-binary participants, but in varied ways for different individuals. For some participants in our study dormant early childhood thoughts of becoming a parent had been rekindled by entry into a same-gender or different-gender partnership that made shared parenthood feasible and desirable. But for other participants LCT principles of agency, life span and linked lives worked differently since parenthood was not contingent upon partnership. Instead parenthood was envisaged as a distinct personal project with single parenting (albeit surrounded by supportive others). Extended family support for having children was mentioned by some focus group participants in conjunction with their parenthood plans, but this was seen as desirable rather than a necessary prerequisite. Nonetheless, participants were mindful of views within their wider family with some participants pointing out that members of their extended family would be less supportive of adoption than they would of genetically related parenthood, which in turn influenced their own preference for exploring fertility treatment.

Planning for parenthood involved participants weighing up whether they (on their own, or in conjunction with other linked lives) had sufficient access to the financial and accommodation resources that children needed: Were they secure in their occupational career pathway? Did they have the right type of home for a child? Here, as in previous studies that focused on (cisgender) LGB and heterosexual people, an intention to have children might be put on hold when career plans were being pursued (Umberson et al., 2010; Bergstrom-Lynch, 2016). In fact, economic considerations potentially seemed to loom larger for the transgender and non-binary people interviewed in our study than they apparently had for participants in other studies. One reason for this was that the financial costs of accessing ART were often higher for those undergoing a physical transition because of the need to budget for cryopreservation of their own cells (Tornello and Bos, 2017; Riggs and Bartholomaeus, 2018; Marinho et al., 2020). Furthermore, participants were having to make decisions about cryopreservation in their late teens and early twenties while still at college or just as their career was beginning with limited financial reserves. In the United Kingdom gender clinics have begun to open up fertility discussions and prepare leaflets to direct clients to fertility services. However, unlike the UK National Health Service funding of gamete extraction and storage prior to cancer treatment, public funding was not generally available for those seeking services for reasons of gender transition. Thus, hopes were raised but then dashed by lack of funding.

### Strengths and Limitations

Undoubtedly, the findings derived from our study remain limited by the small number of participants sampled most of whom were white, middle class, people without disabilities. Our recruitment was through a community organization and those who attended the focus groups came from in and around a large capital city (although some had moved to London from other parts of the United Kingdom and Ireland). Thus, we note that participants might perhaps have been more aware of, and empowered to voice, a transgender and gender diverse equality rights agenda in a community group setting, than if they had been recruited in other ways, for instance via gender identity clinics. In particular, we emphasize a caution that our restricted sampling limited consideration of parenthood by transgender and non-binary people who were assigned male at birth and we would recommend further research specifically aimed at this group.

Notwithstanding the limitations above, our small sample size facilitated an in-depth consideration of the qualitative data gathered to interpret thematic patterns within the data and not simply label content domains (Braun and Clarke, 2013). Furthermore, the sample encompassed people who had not had any hormonal or surgical interventions in relation to gender fulfilment, others who had been prescribed hormones, and those who had undertaken surgery of various kinds. Through the conversations generated in the focus groups we also glimpsed the development of a range of different viewpoints within the transgender and non-binary communities (Vicsek, 2010) and factors, such as the wide age range of participants with different personal circumstances, highlighted cohort and contextualizing factors within the group. The views presented in the groups varied both in favor of future parenthood, or in favor of remaining childfree; thus, we were pleased to have facilitated a safe space for a face-to-face exchange of information and thoughts (Wilkinson, 1999). In addition, our findings are based upon an independent audit and the consideration of focus group data from three separate groups, one of which provided an opportunity for some verification of the preliminary findings from thematic analysis of the first two focus groups.

## Conclusion

Our mixed focus groups of transgender and non-binary people have highlighted the complexity of issues faced by transgender and non-binary people living beyond cisnormativity who delineated an interwoven set of life course considerations in deciding whether to try for parenthood or remain childfree. While considerations of gender identity were involved in plans for parenthood or remaining childfree, it was also apparent that considerations of parenthood or not had reciprocal implications for the realization of gender identity. The challenges of parenthood emphasized by transgender participants were first and foremost medical or societal compounded by possible ignorance, discrimination, and prejudice, for example, in the absence of appropriate funding for fertility treatment or anticipated difficulties in being approved for adoption. Non-binary people highlighted the social challenges they faced in achieving recognition of their gender fluid or gender neutral parenting intentions. Our findings highlight the need for more open discussion, both within the transgender and non-binary community and among professionals working in these fields, of the possibilities of fertility preservation after hormonal or surgical treatments and also of the opportunities for transgender and non-binary people to foster or adopt children.

## Data Availability Statement

The datasets for this article are not publicly available in order to protect the participants’ confidentiality. Requests to access the datasets should be directed to FT, f.tasker@bbk.ac.uk.

## Ethics Statement

The studies involving human participants were reviewed and approved by the Department of Psychological Sciences Research Ethics Committee of Birkbeck, University of London, United Kingdom. The participants provided their written informed consent to participate in this study.

## Author Contributions

FT and JG designed the study, constructed the focus group interview schedule, and collected and curated the data. FT conducted the initial analysis of the data and led to write up of the manuscript which was refined by JG.

## Conflict of Interest

The authors declare that the research was conducted in the absence of any commercial or financial relationships that could be construed as a potential conflict of interest.
